# The time course of different neuromuscular adaptations to short-term downhill running training and their specific relationships with strength gains

**DOI:** 10.1007/s00421-022-04898-3

**Published:** 2022-02-18

**Authors:** Bastien Bontemps, Mathieu Gruet, Julien Louis, Daniel J. Owens, Stella Miríc, Robert M. Erskine, Fabrice Vercruyssen

**Affiliations:** 1 Laboratoire IAPS (N°201723207F), Université de Toulon, Toulon, France; 2 LAMHESS, Université Côte d’Azur, Nice, France; 3 School of Sport and Exercise Sciences, Liverpool John Moores University, Liverpool, UK; 4 Institute of Sport, Exercise and Health, University College London, London, UK

**Keywords:** Eccentric training, Strength, Hypertrophy, Muscle architecture, Aerobic capacity

## Abstract

**Purpose:**

Due to its eccentric nature, downhill running (DR) training has been suggested to promote strength gains through neuromuscular adaptations. However, it is unknown whether short-term chronic DR can elicit such adaptations.

**Methods:**

Twelve untrained, young, healthy adults (5 women, 7 men) took part in 4 weeks’ DR, comprising 10 sessions, with running speed equivalent to 60–65% maximal oxygen uptake ($\dot{V}$O_2max_, assessed at weeks 0 and 4). Isometric and isokinetic knee-extensor maximal voluntary torque (MVT), *vastus lateralis* (VL) muscle morphology/architecture (anatomical cross-sectional area, ACSA; physiological CSA, PCSA; volume; fascicle length, *L*_f_; pennation angle, PA) and neuromuscular activation (VL EMG) were assessed at weeks 0, 2 and 4.

**Results:**

MVT increased by 9.7–15.2% after 4 weeks (*p* < 0.01). VL EMG during isometric MVT increased by 35.6 ± 46.1% after 4 weeks (*p* < 0.05) and correlated with changes in isometric MVT after 2 weeks (*r* = 0.86, *p* = 0.001). VL ACSA (+2.9 ± 2.7% and +7.1 ± 3.5%) and volume (+2.5 ± 2.5% and +6.6 ± 3.2%) increased after 2 and 4 weeks, respectively (*p* < 0.05). PCSA (+3.8 ± 3.3%), PA (+5.8 ± 3.8%) and *L*_f_ (+2.7 ± 2.2%) increased after 4 weeks (*p* < 0.01). Changes in VL volume (*r* = 0.67, *p* = 0.03) and PCSA (*r* = 0.71, *p* = 0.01) correlated with changes in concentric MVT from 2 to 4 weeks. $\dot{V}$O_2max_ (49.4 ± 6.2 vs. 49.7 ± 6.3 mL·kg^−1^·min^−1^) did not change after 4 weeks (*p* = 0.73).

**Conclusion:**

Just 4 weeks’ moderate-intensity DR promoted neuromuscular adaptations in young, healthy adults, typically observed after high-intensity eccentric resistance training. Neural adaptations appeared to contribute to most of the strength gains at 2 and 4 weeks, while muscle hypertrophy seemed to contribute to MVT changes from 2 to 4 weeks only.

## Introduction

Muscle strength is a major contributor to quality of life and physical performance in both clinical and sporting contexts. In this regard, traditional eccentric-based interventions (e.g. resistance training) are particularly relevant for enhancing muscle strength (for review, Douglas et al. 2017a) and are becoming increasingly widespread in rehabilitation, strength training or injury prevention programmes for athletes and patients. From a mechanical point of view, eccentric muscle contraction refers to a muscle action that occurs when the magnitude of the force applied to the muscle exceeds the momentary force produced by the muscle itself, resulting in a lengthening action of the musculotendinous system (Lindstedt et al. 2001; Douglas et al. 2017b). These unique mechanical properties typically lead to a lower metabolic cost (Abbott et al. 1952; Fenn 1924; Hody et al. 2019) and lower cardiorespiratory demands (Chasland et al. 2017; Dufour et al. 2004; Penailillo et al. 2017; Lemire et al. 2021) for a matched workload compared to other contraction modalities, which is of paramount importance in athletic and clinical populations.

It is well documented that chronic use of resistance (both concentric and eccentric) exercise increases muscle strength through neural and muscle morphological adaptations (for review, Douglas et al. (2017a). Indeed, eccentric training is a potent stimulator of strength gains (Seynnes et al. 2007; Franchi et al. 2014), and is likely more efficient to promote muscle strengthening than concentric or isometric training (Douglas et al. 2017a; Roig et al. 2009). However, more ecologically valid modes of eccentric exercise (e.g. variable eccentric exercises, such as downhill walking/running) might also promote strength gains in a context that is more relevant to daily and/or athletic activity (Vikne et al. 2006). Variable eccentric exercises are characterised by repetitive low-to-moderate-intensity eccentric contractions (i.e. *low-intensity*, *high-volume* eccentric exercise) with minimal range of motion, compared to traditional eccentric modalities that typically involve fewer repetitions but a much higher load (e.g. flywheel/resistance exercise). Downhill running (DR) is a variable eccentric modality (i.e. endurance-like eccentric exercise) that could stimulate locomotor’s muscle function, while simultaneously challenging the cardiorespiratory system. Importantly, DR has become more prevalent in athletic settings due to the growing interest in running events including notable negative elevations (e.g. trail running races, mountain running events, as well as some specific road races). In this regard, implementing DR sessions into a runner’s training routine might be an effective way to improve strength and power, which are important for improving endurance performance (Blagrove et al. 2018), while simultaneously improving or maintaining aerobic capacity, all the while being specific to the athlete’s sport, i.e. running (Bontemps et al. 2020). However, in a study comparing short-term (5 weeks’), moderate-intensity level running with DR in untrained, young adults, maximal oxygen uptake ($\dot{V}$O_2max_) increased after level running but not DR, while maximum knee-extensor strength increased after DR but not level running (Toyomura et al. 2018). Thus, DR and level running appear to elicit different adaptations to the neuromuscular and aerobic systems but how DR leads to gains strength (but not $\dot{V}$O_2max_) after such a short period of training is not known.

To the best of our knowledge, only three studies have investigated the effects of DR training on strength gains and the results are contrasting. Although Law et al. (1995) reported no strength improvement following 8 weeks’ DR training (one session/week, 30 min at − 10%, running speed equivalent to 50–60% $\dot{V}$O_2peak_) in military-trained individuals, Toyomura et al. (2018) reported increases in isometric and isokinetic maximal voluntary torque (MVT) after 5 weeks’ DR training (three sessions/week, 20 min at − 10%, running speed equivalent to lactate threshold, i.e. ~ 69% $\dot{V}$O_2peak_) in untrained participants. In addition, Theofilidis et al. (2021) did not observe an increase in isometric or isokinetic MVT in either the knee extensors or flexors following 8 weeks’ DR training (two sessions/week, 10 × 30 s bouts at − 10% slope, interspersed with 60-s rest intervals, and running speed equivalent to 90% of the speed associated with $\dot{V}$O_2max_). However, the authors did observe that rate of torque development (measured during the isometric MVT) improved after the DR training. Although the discrepancies between studies may partly be explained by DR training characteristics (e.g. volume) and/or participants’ initial training status, the question remains: can DR training induce significant muscle strengthening to the lower limb extensor muscles and, if so, what are the main physiological factors contributing to these functional changes? Since the muscle hypertrophic response to overloading is believed to be mediated by mechanical tension, exercise-induced muscle damage and/or metabolic stress (Schoenfeld 2010), chronic and/or repeated use of DR exercise could be an interesting strategy to promote muscle strength gains via neuromuscular adaptations, while potentially enhancing or maintaining $\dot{V}$O_2max_. However, to date, it is not known whether chronic neural and muscle morphological adaptations occur in response to short-term (e.g. 4–5 weeks’) DR training. Such information would allow clinicians, coaches, and athletes to re-consider short-term chronic training possibilities, using more ecologically valid methods of training. Moreover, it may help to further our understanding of how different modes, intensities and volumes of eccentric exercise (e.g. DR compared to traditional resistance exercise) can potentially induce similar neuromuscular adaptations over a relatively short period of time.

The purpose of the present study was to examine the time course (over 0, 2 and 4 weeks) of change in the functional, neural, morphological and architectural adaptations of the quadriceps femoris in response to short-term, moderate-intensity DR training in untrained, young adults. We hypothesised that DR training would lead to rapid neural and muscle hypertrophic adaptations due to the high volume of eccentric loading, with no effect on $\dot{V}$O_2max_. Moreover, we hypothesised that neural changes would be more strongly related to the changes in knee-extensor strength (particularly during the first 2 weeks of training), while changes in quadriceps muscle size would occur during the latter half of the training period and would be related to changes in strength during this period.

## Materials and methods

### Ethics statement

This study was approved by Liverpool John Moores University Research Ethics Committee (19/SPS/024) and conformed to the standards of use of human participants in research as outlined in the Sixth Declaration of Helsinki. All participants were informed of the experimental procedures and gave their written informed consent before any testing was conducted.

### Participants

Fifteen participants volunteered to take part in the study but three withdrew during the study for personal reasons. A total of 12 adults (5 females and 7 males; age: 25.1 ± 4.9 years; height: 1.69 ± 0.08 m; mass: 66.7 ± 13.1 kg; BMI: 23.2 ± 3.3 kg·m^−2^; fat mass: 19.7 ± 7.4%) completed the 4-week DR training programme. A minimal sample size was estimated prior to conduct the study with G*Power software (v3.1.9.6, Heinrich-Heine-Universität Düsseldorf, Düsseldorf, Germany). Briefly, the estimation was performed using the eccentric strength gains observed after 5 weeks’ DR training (Toyomura et al. 2018), and results from our a priori power calculation deemed a minimum of nine participants was necessary to detect an effect of DR (One-tailed *t* test; α: 0.05; Power: 0.8). All participants were healthy and recreationally active individuals (determined by responses to a standardised readiness to exercise questionnaire, in which they reported that they performed one to three low-to-moderate-intensity endurance training sessions as part of their weekly exercise routine). Participants were free from any medical contraindications and had no history of musculotendinous injuries, or plyometric, eccentric and/or heavy resistance training in the 6 months prior to the study. They had also never performed any DR-specific training. Further, they were asked to maintain habitual lifestyle habits and physical activity for the duration of the study.

### Study design

**Fig. 1:**
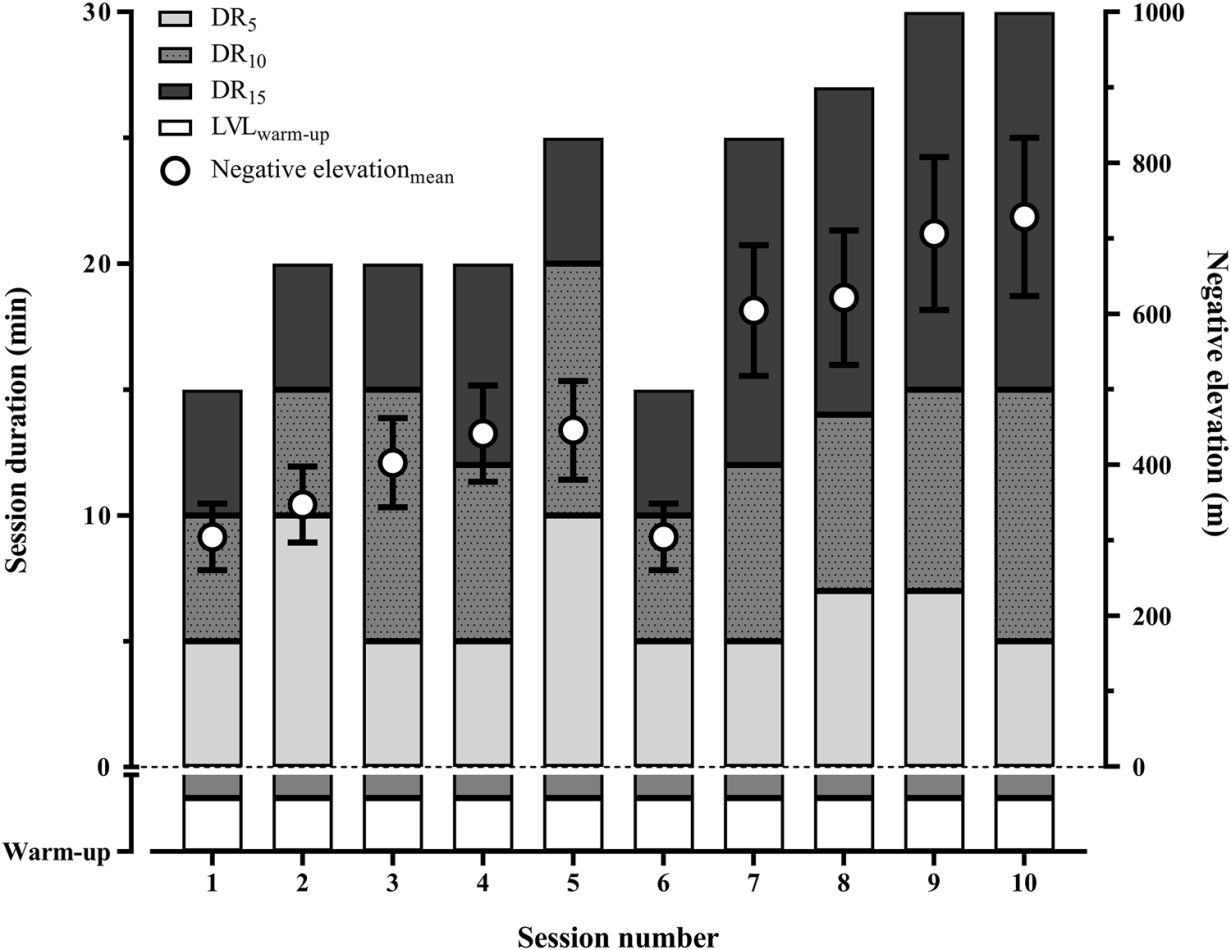
Schematic overview of the downhill running (DR) training programme. Bars represent each separate DR training session, split into the three negative slopes (− 5%, − 10% and − 15%; i.e. DR_5_, DR_10_ and DR_15_, respectively). White dots represent the mean ± SD negative elevation for each separate DR training session (excluding warm-up)

Participants were required to visit the laboratory on 13 separate occasions (Fig. 1). During the first visit, participants performed a maximal running test to exhaustion to determine maximal oxygen uptake ($\dot{V}$O_2max_). Following a 20-min passive recovery period, participants were familiarised with DR at three different slopes (− 5%, − 10% and − 15%; i.e. DR_5_, DR_10_ and DR_15_, respectively) at grade-related speeds associated with 60–65% $\dot{V}$O_2max_ (i.e. $\dot{V}$O_2_ was measured at all grades) for 10 to 15 min, as well as with all other experimental procedures. The subsequent visits were allocated to DR training and/or testing sessions (Fig. 1). The 13th visit was used to determine post-training $\dot{V}$O_2max_. Laboratory conditions remained stable throughout the training sessions (temperature: 23.4 ± 1.0 °C; relative humidity: 41.7 ± 7.4%).

### Training programme overview

A total number of 10 training sessions were performed by all participants under the constant supervision of B.B. and S.M. for a period of four consecutive weeks, with 1–2 days’ rest allocated between training sessions. Participants were required to keep the same training schedule over the programme (± 1.5 h). All DR training bouts comprised consecutive treadmill running at DR_5_, DR_10_ and DR_15_ at a speed associated with a metabolic intensity of 60–65% $\dot{V}$O_2max_ on each grade. Based on pilot tests, this metabolic intensity was selected to ensure that participants maintained moderate and tolerable running speeds on prolonged treadmill runs. Grade-related speeds were estimated from the familiarisation session (where $\dot{V}$O_2_ was measured at all grades to ensure running speeds corresponded to 60–65% $\dot{V}$O_2max_) and readjusted if necessary during the first training session to keep speeds consistent throughout the training programme (8.4 ± 0.9, 10.3 ± 1.0, 11.8 ± 1.8 and 13.0 ± 1.8 km·h^−1^ for the level grade, DR_5_, DR_10_ and DR_15_, respectively). Total running time (from 15 to 30 min) and/or training load (by increasing running time at DR_10_ and DR_15_ to increase relative negative elevations) gradually increased throughout the 4-week training period. Before each DR training session, participants carried out a warm-up, comprising 7 min’ level running and 3 min’ DR_10_ at a speed associated with a metabolic intensity of 60–65% $\dot{V}$O_2max_.

### Experimental protocol

DR training-induced neuromuscular adaptations and their respective time course of changes were evaluated at 0 weeks (i.e. before starting the DR training), 2 weeks (i.e. before starting training session #6) and 4 weeks (i.e. 3–5 days after the completion of the 10th and final DR training bout). During those testing sessions, the following physiological assessments of the right leg were performed in this order: *vastus lateralis* (VL) muscle morphology and architecture, knee-extensor (KE) isometric and isokinetic MVT, followed by neuromuscular activation. $\dot{V}$O_2max_ was assessed at 0 weeks and 4 weeks.

#### KE isometric, eccentric and concentric MVT

KE isometric, eccentric and concentric MVT (MVT_ISO_, MVT_ECC_, and MVT_CON_) were assessed in a non-fatigued condition using an isokinetic dynamometer (Humac Norm, CSMI, Massachusetts, USA), with the hip joint set at 85° (supine = 180°) and the participant’s chest, waist and thigh secured to the chair with inextensible straps. The dynamometer was calibrated, gravity corrected, and all settings were individually recorded and reused for subsequent visits. The warm-up comprised 10 concentric repetitions (30°·s^−1^) performed with increasing intensity, i.e. ~ 10% to ~ 80% of perceived maximum effort, followed by two repetitions at ~ 80% isometric MVT. Thereafter, participants had to perform three repetitions of MVT_ECC_ and MVT_CON_ (both at 30°·s^−1^; range of motion: 0 to 110°, with 0° corresponding to full knee extension) interspersed by 5-s rest, and contraction modalities were separated by three 3 min of passive rest. This was followed by four MVT_ISO_ (each lasting ~ 3 to 4 s) with the knee joint set at 90° knee flexion, interspersed by 30-s passive rest. Real-time visual and verbal feedback regarding torque production, as well as verbal encouragement, were provided throughout the test. For all contraction modalities, the repetition with the highest MVT (MVT_peak_) was used for subsequent analysis.

#### Voluntary activation and electromyographic activity

The interpolated twitch technique (ITT) was conducted with transcutaneous electrical stimuli (100 Hz doublet) delivered to the right femoral nerve via a constant-current stimulator (DS7A, Digitimer, Welwyn Garden City, Hertfordshire, UK). A 15-mm diameter cathode (Contrôle Graphique, Brie‐Comte‐Robert, France) was pressed manually by the investigator onto the femoral triangle, and a 50 mm × 90 mm rectangular anode (Durastick Plus, DJO Global, Vista, CA, USA) was attached to the right gluteal fold. The precise location of the cathode was electrically determined (within three to five attempts) using single square wave pulses (200 μs duration) as the position that evoked the greatest single twitches and concomitant evoked compound action potential (*M*-wave) response for a particular submaximal electrical current (typically 30–50 mA). The femoral nerve was then stimulated in a relaxed state with 75 mA pulses of 200 μs, and this was incrementally increased by 10 or 25 mA until no further increase in torque was observed (typically 80–180 mA). This amplitude was increased by 30% to ensure supramaximal stimulation during the ITT 2 min later, for which one doublet (*d*) was superimposed on the plateau of a MVC_ISO_, and one (control doublet, *D*) two seconds after cessation of the MVC_ISO_. These procedures were performed on the last two of the four MVT_ISO_. Voluntary activation capacity (VA, %) was calculated according to the following equation: VA% = 100 × (1 − (*d* × *h*)/*D*), where *d* is the superimposed doublet torque, *h* the ratio between the torque at stimulation time and peak MVT, and *D* is the control doublet torque (Strojnik and Komi 1998).

#### Surface electromyography (EMG)

Surface EMG activity was recorded from the right VL during all MVT_ISO_ and during evoked contractions using surface bipolar electrodes (Ag–AgCl, Blue Sensor N-00-S, Ambu, Denmark). Following preparation of the skin (shaving, lightly abrading and cleansing with 70% ethanol), two electrodes were attached (20 mm apart) on the skin at a location corresponding to the distal third of the muscle length along the mid-sagittal plane (according to the SENIAM recommendations; http://www.seniam.org/), and in the direction of the muscle fascicles (identified using ultrasound, as detailed below). The reference electrode was placed on the skin over the right patella. All electrode locations (with regard to distances from anatomical landmarks) were measured and recorded for relocation during subsequent tests. Surface EMG signals were amplified (100×, differential amplifier 20–450 Hz) and sampled at 2 kHz with the same analogue-to-digital converter (MP150 BIOPAC Systems, Inc., Santa Barbara, USA) and PC as the torque signal, prior to being band-pass filtered in both directions between 10 and 500 Hz. The root mean square (RMS) of the EMG signal over a 300 ms epoch around peak MVT_ISO_ (± 150 ms) was used to assess VL activation.

#### Evoked compound muscle action potential (M-wave)

To further minimise the variability in VL EMG RMS amplitude (Erskine et al. 2014), EMG measured during the first two MVT_ISO_ was normalised to the evoked and potentiated supramaximal *M*-wave (*M*_max_) in the VL. To elicit *M*-waves from the VL, the femoral nerve was electrically stimulated (DS7AH, Digitimer Ltd., Welwyn Garden City, UK) with single square wave pulses (200 μs duration), using the same cathode and anode setup as used during the ITT. Two supramaximal and potentiated M-waves were evoked with the muscle at rest 2 and 4 s after a MVT_ISO_. *M*_max_ was defined as the mean peak-to-peak EMG response to these two stimuli. The mean of the two was used for subsequent analysis.

#### Ultrasonographic measurements

Muscle morphological and architectural measurements of the VL were obtained using a real-time B-mode ultrasonography system (Philips EPIQ Elite, Amsterdam, The Netherlands) with a 7.5 MHz 40-mm-wide linear-array probe. The VL muscle is the largest of the quadriceps femoris heads (Erskine et al. 2009), and the morphological and architectural assessments of the VL muscle are highly reproducible (Monti et al. 2020; Reeves et al. 2004a; Noorkoiv et al. 2010a; Noorkoiv et al. 2010b). Measurements were conducted on the right leg while the participant was resting supine on an examination bed with the knee fully extended and the ankle fixed in the neutral position. All images were taken after 15 min of rest to avoid fluid shifts that might induce interstitial and/or intracellular changes (Berg et al. 1993). During this period, regions of interest were identified through ultrasonographic measurements, then marked on the skin using a permanent marker pen. A water-soluble gel was generously applied to the transducer to aid acoustic coupling and to limit pressure applied to the skin by the transducer. At least three images were taken from each participant for the analysis of the different variables, and the mean of the best three was used for subsequent analysis. All ultrasound images were analysed using ImageJ software (National Institutes of Health, Bethesda, MA, USA). Due to a technical issue encountered at week 2 for one participant, the sample number regarding morphological muscle adaptations at 2 weeks was *n* = 11.

Muscle thickness (MT), fascicle pennation angle (PA) and fascicle length (*L*_f_) were measured with the transducer placed along the mid-sagittal plane of the VL, perpendicular to the skin and carefully aligned to the direction of the fascicles. MT was defined as the perpendicular distance between superficial and deep aponeuroses (Narici 1999), while PA was defined as the angle the fascicles inserted into the muscle’s deep aponeurosis (Gans 1982; Kawakami et al. 1993). Ultrasound images were captured at 50% VL muscle length (i.e. from the distance between proximal and distal musculotendinous junctions) and 50% VL width (i.e. from the distance between the mediolateral boundaries of the VL) for MT and PA. In contrast, *L*_f_ was determined using extended-field-of-view (EFOV) ultrasound images from 20 to 80% of VL muscle length (along the mid-sagittal plane) to identify the fascicular paths between the insertions of the fascicles into the superficial and deep aponeuroses. It should be emphasised that while *L*_f_ consistently passed through 50% muscle length, at least three fascicles were measured along the length of the muscle (and the mean of the three was used to determine *L*_f_), thus *L*_f_ incorporated fascicles from the distal, central, and proximal regions of the VL. Transducer velocity was kept constant during EFOV acquisition.

Anatomical cross-sectional area (ACSA) of VL muscle was measured with the transducer moving along the transversal plane of the VL at three different locations: 25%, 50% and 75% muscle length (ACSA_25%ML_, ACSA_50%ML_ and ACSA_75%ML_). As above, transducer velocity was kept constant during EFOV acquisition. Muscle volume was then calculated using the truncated cone formula as previously reported for ultrasonography use (Esformes et al. 2002) (see formula):

$$\mathrm{Muscle \ volume}=\frac{1}{3} h \left(a+\sqrt{\left(ab\right)+b}\right),$$

where *h* corresponds to the distance between two ACSAs, *a* and *b* correspond to the ACSAs of the muscle in the two scans. The volume of the entire muscle was calculated by summing up all the inter-scan muscular volumes.

VL physiological cross-section area (PCSA) was calculated by dividing VL muscle volume by *L*_f_.

### Test–retest reproducibility of MVT, voluntary activation, and ultrasonographic measurements

Test–retest reproducibility for MVTs and VA was determined on seven participants (1 female and 6 males; age: 25.3 ± 2.7 years; height: 1.73 ± 0.05 m; body mass: 72.6 ± 11.2 kg; BMI: 24.1 ± 3.1; fat mass: 18.9 ± 9.4%) and on six participants for ultrasonographic measurements (2 females and 4 males; age: 22.9 ± 1.5 years; height: 1.74 ± 0.06 m; mass: 68.2 ± 8.9 kg; BMI: 22.6 ± 2.2; fat mass: 20.0 ± 6.7%). All measurements were performed by the same investigator (BB). Inter-day reproducibility (interspersed by 24 to 72 h) was expressed as coefficient of variation (CV) and intraclass correlation coefficient (ICC, model: two-way mixed; type: absolute agreement) with 95% confidence intervals (CIs). In addition, to rule out the potential bias of repeated measurements on morphological muscle adaptations to training, the minimum detectable changes were calculated (Table 1) (Weir 2005). For all variables, CVs were low (except for MVT_ECC_), and ICCs were high (≥ 0.80) with narrow CIs (ICC 95% CI: 0.78 to 0.99) except for VA (ICC 95% CI: 0.32 to 0.97). It should be noted that the CV and ICC CIs for MVT_ECC_ and VA, respectively, were likely increased by just a few participants’ deviating test–retest data having a relatively greater influence due to the moderate sample size.

**Table 1:** Test–retest reproducibility of knee-extensor muscle strength, voluntary activation (VA, i.e. interpolated twitch technique) and *vastus lateralis* muscle morphology measurements in seven recreationally active participants

	Minimum detectable change (95% CI)	CV (%)	ICC (95% CI)
MVT_ISO_	1.27% (± 3.88 N·m)	3.613	0.995 (0.969–0.999)
MVT_ECC_	5.90% (± 16.90 N·m)	12.454	0.929 (0.648–0.987)
MVT_CON_	2.79% (± 6.23 N·m)	5.744	0.961 (0.809–0.993)
VA	2.42% (± 2.10%)	4.830	0.817 (0.318–0.965)
ACSA_mean_	1.04% (± 0.22 cm^2^)	1.083	0.996 (0.974–0.999)
ACSA_75%ML_ (proximal)	1.76% (± 0.35 cm^2^)	1.341	0.987 (0.888–0.999)
ACSA_50%ML_ (medial)	1.44% (± 0.37 cm^2^)	2.189	0.993 (0.957–0.999)
ACSA_25%ML_ (distal)	1.62% (± 0.28 cm^2^)	1.704	0.993 (0.903–0.999)
Muscle volume	2.63% (± 7.30 cm^3^)	2.700	0.990 (0.936–0.999)
PCSA	2.10% (± 1.13 cm^2^)	2.634	0.987 (0.905–0.998)
Fascicle length	0.87% (± 0.67 mm)	1.062	0.990 (0.940–0.999)
Fascicle pennation angle	0.67% (± 0.12°)	2.056	0.978 (0.848–0.997)
Muscle thickness	2.14% (± 0.49 mm)	2.710	0.966 (0.782–0.995)

Measurements were taken on two separate occasions within 3 days using methods presented in “Materials and methods”

*MVT* maximal voluntary torque; *VA* voluntary activation measured during MVT_ISO_; *ACSA* anatomical cross-sectional area, *PCSA* physiological cross-section area; *p* < 0.001 for all ICCs

### Maximal oxygen uptake

Participants performed a maximal running test to exhaustion on a motorised treadmill (HP Cosmos, Nussdorf, Germany) to determine $\dot{V}$O_2max_. The test started with the slope and speed set to +1% and 7 km·h^−1^, respectively. Speed was then increased by 1 km·h^−1^ per min up to 16 km·h^−1^, at which point the slope was increased by 1% per min until exhaustion. Breath-by-breath gas exchange values were recorded and averaged every 15 s by a gas exchange analyser (Oxycon Pro, Jaeger, Hoechberg, Germany). The $\dot{V}$O_2_ was considered maximal ($\dot{V}$O_2max_) when at least two of the three following criteria were met: (1) a levelling off of $\dot{V}$O_2_ with increasing intensity (an increase of no more than 2 mL·kg^−1^·min^−1^); (2) a HR within 10 beats·min^−1^ of the age-predicted maximum (220 minus age in years); (3) a respiratory exchange ratio (RER) greater than 1.05. $\dot{V}$O_2max_ was determined from the mean of the three consecutive highest values over a 30-s interval reached during the last stage of the protocol.

### Statistical analysis

All variables are expressed as mean ± standard deviation. All data were tested for normality using the Shapiro–Wilk’s normality test and sphericity was assumed. A one-way analysis of variance was used to determine the main effect of *training* only (i.e. repeated measures). A two-factor within-subjects ANOVA was also used to determine the main effects of *training* × *location* for VL muscle ACSAs. Mixed-effects models were used, as one data value was missing for one participant. When significant main effects for one-way ANOVAs or *interaction* for two-way ANOVAs occurred, post hoc pairwise comparisons with Bonferroni adjustments were used. Relationships between percentage changes in MVT and percentage changes in independent neuromuscular variables were tested with a Pearson’s product-moment correlation. The coefficient of correlation (r) and significance level were reported for each separate analysis (Table 3). The significance level for all analyses was set at *p* < 0.05. Statistical and post hoc analyses were performed on GraphPad Prism software (version 8.0; GraphPad Software Inc., San Diego, CA, USA).

## Results

### Isometric and isokinetic strength

Main effects of *training* were observed for MVT_ISO_ (*p* < 0.001), MVT_ECC_ (*p* < 0.001) and MVT_CON_ (*p* = 0.001). Post hoc analyses revealed that training-induced changes occurred for MVT_ISO_, MVT_ECC_ and MVT_CON_ at 4 weeks (all, *p* < 0.01; Table 2). MVT increased from baseline to 4 weeks by 9.7 ± 11.2% (*p* < 0.01), 15.2 ± 11.3% (*p* < 0.001) and 12.9 ± 11.8% (*p* < 0.01) for MVT_ISO_, MVT_ECC_ and MVT_CON_, respectively, with no difference between contraction modalities (*p* > 0.05). Only MVT_ECC_ increased significantly from baseline to 2 weeks (+8.6 ± 11.3%; *p* = 0.03).

**Table 2:** Downhill running training-induced changes in knee-extensor muscle strength and *vastus lateralis* muscle morphology. Data are presented as mean ± SD

	Baseline	2 weeks	4 weeks
Isometric MVT (N·m)	228 ± 69	231 ± 83	254 ± 88*
Eccentric MVT (N·m)	240 ± 54	249 ± 80*	268 ± 84*
Concentric MVT (N·m)	179 ± 54	189 ± 61	200 ± 57*
ACSA_mean_ (cm^2^)	20.9 ± 4.3	21.4 ± 4.5*	22.3 ± 4.2*^§^
ACSA_75%ML_ (proximal)	19.9 ± 3.9	20.4 ± 4	21.1 ± 3.9*^§^
ACSA_50%ML_ (medial)	25.7 ± 5.5	26.3 ± 5.7	27.2 ± 5.3*^§^
ACSA_25%ML_ (distal)	17 ± 4.1	17.8 ± 4.3	18.6 ± 4.1*^§^
Muscle volume (cm^3^)	395 ± 91	406 ± 96*	420 ± 92*^§^
PCSA (cm^2^)	52 ± 11.9	53.3 ± 12.5	54.0 ± 12.2*
Fascicle length (mm)	76.5 ± 8.3	76.8 ± 9.2	78.5 ± 8.9*^§^
Fascicle pennation angle (°)	17.5 ± 1.3	17.9 ± 1.1	18.5 ± 1.0*^§^
Muscle thickness (mm)	22.7 ± 3.3	23.4 ± 3.0	23.7 ± 2.7*

*MVT* maximal voluntary torque, *ACSA* anatomical cross-sectional area, *ML* muscle length, *PCSA* physiological cross-sectional area

*Different to baseline values (*p* < 0.05); ^§^different to values at 2 weeks (*p* < 0.05)

### Changes in neuromuscular activation

A main effect of *training* was observed for VL EMG RMS (*p* = 0.04) but not for VA (*p* = 0.68) nor VL EMG RMS/M_max_ (*p* = 0.10). VA values were 84.0 ± 8.9%, 84.2 ± 12.7%, and 86.5 ± 7.3% at 0, 2 and 4 weeks, respectively. Changes in VL EMG RMS (relative to baseline) corresponded to 122.8 ± 37.9% (*p* = 0.18) and 135.6 ± 46.1% (*p* < 0.05) when analysed at 2 and 4 weeks, respectively.

### VL muscle morphological and architectural adaptations

**Fig. 2:**
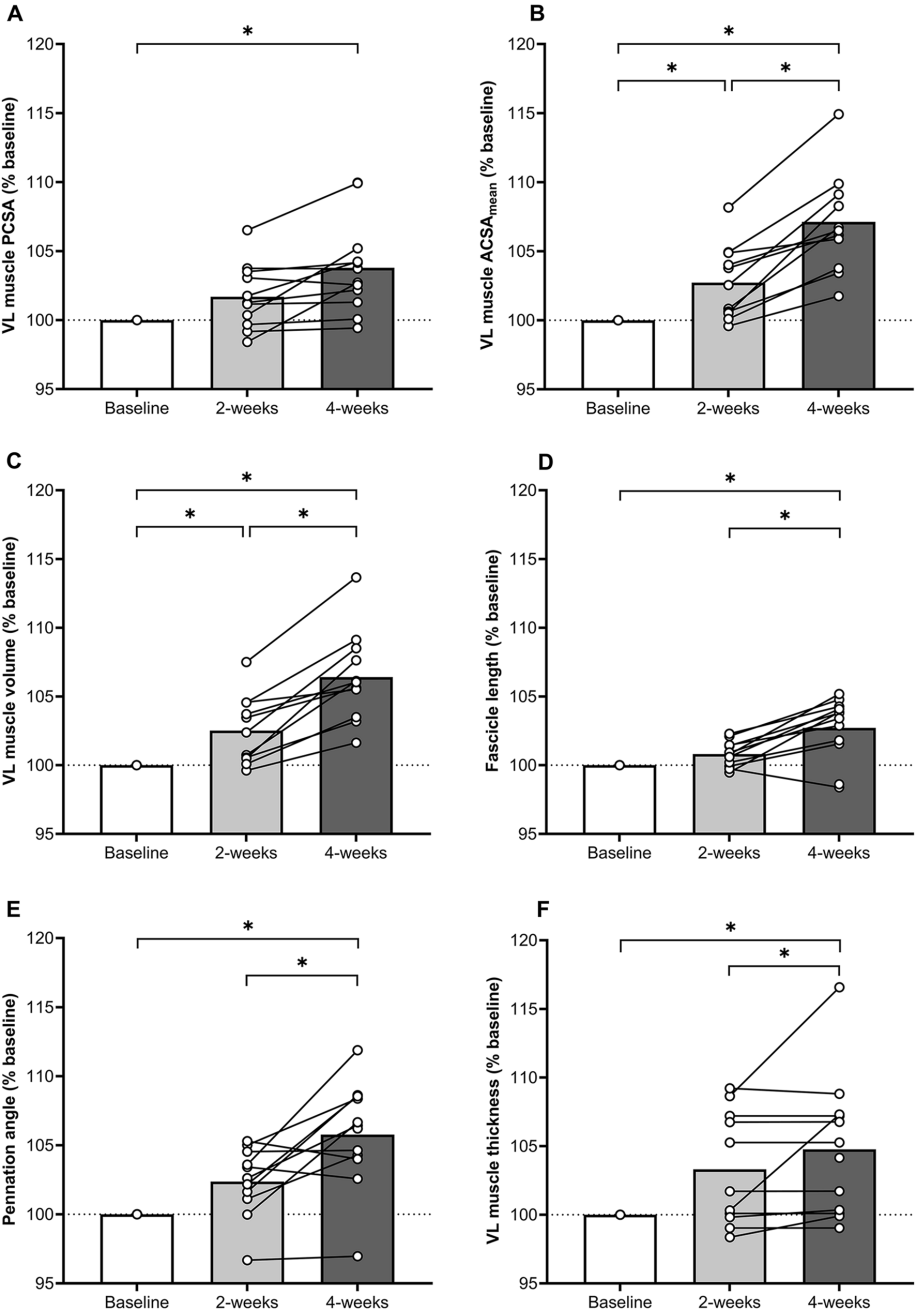
*Vastus lateralis* (VL) morphological and architectural adaptations over the 4-week downhill running (DR) training period. Changes in VL physiological cross-sectional area (PCSA; **A**), mean anatomical cross-sectional area (ACSA_mean_; **B**), volume (**C**), fascicle length (**D**), pennation angle (**E**) and thickness (**F**) are further presented. Grey and black bars represent mean % changes from 0 to 2 weeks’, and from 0 to 4 weeks’ DR training, respectively. Connected white data points represent individual participant changes between time points; *Mean changes differed between time points (*p* < 0.05)

A main effect of *training* (all *p* < 0.01) was observed for the following variables: VL MT, PA, *L*_f_, ACSA_mean_, ACSA_25%ML_, ACSA_50%ML_, ACSA_75%ML_, muscle volume and PCSA (Fig. 2, Table 2). For ACSA, a main effect of *training* (*p* < 0.001) and *location* (*p* < 0.001) were observed but no interaction. Post hoc comparisons revealed that the following VL morphological changes occurred at 2 weeks (*p* < 0.01, Fig. 2, Table 2): ACSA_mean_ (+2.9± 2.7%) and VL muscle volume (+2.5± 2.5%) (Fig. 2, Table 2). The following VL morphological changes occurred at 4 weeks from baseline (*p* < 0.01, Fig. 2, Table 2): ACSA_25%ML_ (+10.7 ± 8.0%); ACSA_50%ML_ (+5.9 ± 3.8%); ACSA_75% ML_ (+6.1 ± 4.0%); ACSA_mean_ (+7.1 ± 3.5%); muscle volume (+6.6 ± 3.2%); PCSA (+3.8 ± 3.3%); MT (+4.8 ± 5.0%); PA (+5.8 ± 3.8%); and *L*_f_ (+2.7 ± 2.2%).

### Relationships between strength gains and neuromuscular adaptations

All bivariate correlations are presented in Table 3. Regarding percentage changes in MVT_ISO_, significant correlations were observed with percentage changes in VL EMG RMS from 0 to 2 weeks, VL RMS/M_max_ from 2 to 4 weeks, and with percentage changes in VA and VL RMS/M_max_ from 0 to 4 weeks. While no significant correlations were observed between percentage changes in MVT_ECC_ and neural parameters investigated, a significant correlation was observed between percentage changes in MVT_CON_ and VA (*r* = 0.63; p = 0.03) from 2 to 4 weeks. Regarding percentage changes in MVT (all contraction types) and muscle morphological adaptations, only changes in MVT_CON_ correlated with changes in VL PCSA and volume, both from 2 to 4 weeks only (Table 3).

**Table 3:** Correlations between downhill running (DR) training-induced changes in knee-extensor muscle strength and different neuromuscular adaptations

	MVT_ECC_ (N∙m)			MVT_ISO_ (N∙m)			MVT_CON_ (N∙m)		
	0 to 2 weeks	2 to 4 weeks	0 to 4 weeks	0 to 2 weeks	2 to 4 weeks	0 to 4 weeks	0 to 2 weeks	2 to 4 weeks	0 to 4 weeks
VA, %	*r* = − 0.11	*r* = 0.51	*r* = − 0.07	*r* = 0.15	*r* = − 0.28	***r* = 0.83**	*r* = 0.34	***r* = 0.63**	*r* = − 0.25
	*p* = 0.74	*p* = 0.11	*p* = 0.82	*p* = 0.63	*p* = 0.37	***p* < 0.001**	*p* = 0.27	***p* = 0.03**	*p* = 0.44
VL EMG RMS, mV	**–**	**–**	**–**	***r* = 0.86**	*r* = 0.03	***r* = 0.53**	**–**	**–**	**–**
				***p* < 0.001**	*p* = 0.92	***p* = 0.08**			
VL EMG RMS/M_max_, mV	**–**	**–**	**–**	*r* = 0.004	***r* = 0.65**	***r* = 0.67**	**–**	**–**	**–**
				*p* = 0.92	***p* = 0.03**	***p* = 0.08**			
PCSA, cm^2^	*r* = − 0.10	*r* = 0.22	*r* = − 0.01	*r* = 0.49	*r* = 0.12	*r* = 0.18	*r* = − 0.35	***r* = 0.71**	*r* = − 0.23
	*p* = 0.79	*p* = 0.54	*p* = 0.97	*p* = 0.12	*p* = 0.72	*p* = 0.59	*p* = 0.30	***p* = 0.01**	*p* = 0.47
Volume, cm^3^	*r* = − 0.19	*r* = 0.28	*r* = − 0.15	*r* = 0.36	*r* = 0.38	*r* = 0.06	***r* = **− **0.53**	***r* = 0.67**	*r* = − 0.17
	*p* = 0.61	*p* = 0.42	*p* = 0.65	*p* = 0.28	*p* = 0.24	*p* = 0.86	***p* = 0.10**	***p* = 0.03**	*p* = 0.60

*MVT* maximal voluntary torque, *VA* voluntary activation measured during MVT_ISO_, *VL RMS* root mean square electromyographic signal of the *vastus lateralis* (VL) measured during MVT_ISO_, *M*_*max*_ maximal M-wave amplitude, *ACSA* VL anatomical cross-sectional area, *PCSA* VL physiological cross-sectional area

### Maximal oxygen uptake

No pre- to post-training difference was observed regarding $\dot{V}$O_2max_ (*p* = 0.73). $\dot{V}$O_2max_ values corresponded to 49.4 ± 6.2 mL·min^−1^·kg^−1^ and 49.7 ± 6.3 mL·min^−1^·kg^−1^ at 0 and 4 weeks, respectively.

## Discussion

The aim of the present study was to examine the time course of change in the functional, neural, morphological and architectural adaptations of the quadriceps femoris in response to short-term, moderate-intensity DR training in untrained, young adults. After the 4-week DR training period, peak knee-extensor MVT increased by 9.7–15.2% for all three contraction types (eccentric, isometric and concentric). These strength gains were accompanied by significant changes in VL EMG activity, muscle morphology, i.e. increases in VL muscle PCSA, volume and ACSA_mean_, and architecture, i.e. increases in VL PA and *L*_f_. However, after just 2 weeks of DR training, significant gains in muscle strength (MVT_ECC_) and VL morphology (ACSA_mean_ and volume) were identified. Strength gains (especially MVT_ISO_) were predominantly related to neural adaptations at both 2 and 4 weeks’ DR training. Muscle hypertrophy was related to changes in concentric strength in the second half of the DR training period only. However, DR training did not enhance maximal oxygen uptake. Taken together, these novel results suggest that short-term, moderate-intensity DR training is an effective method of promoting rapid gains in knee-extensor muscle strength, size and structure. The former appears to be explained mainly by neural changes (in the first and second half of the training period) and to a smaller extent by muscle hypertrophy (in the second half of the training period only).

Increases in knee-extensor muscle strength (all three contraction types) were observed following 4 weeks’ DR training (+9.7–15.2%) in previously untrained, healthy adults. This result is consistent with Toyomura et al. (2018), who reported an improvement in isometric and isokinetic knee-extensor MVT (+9–24%) at multiple angular velocities following 5 weeks’ DR training, in previously untrained, healthy young men. Thus, short-term DR training can be considered a potent stimulator of knee-extensor strength gains. Short-term DR training may, therefore, have implications for both clinical and athletic contexts (e.g. by improving health and balance in clinical populations, and increasing physical performance in athletic populations), thus providing a safe and effective alternative method (to resistance training) for inducing strength gains. However, this remains to be verified specifically in those populations. Interestingly, our results showed a faster time course for gains in eccentric vs. isometric and concentric strength, with detectable changes observed at 2 weeks, while isometric and concentric strength increased only at 4 weeks. Since muscle strength improvement has been suggested to be training specific (Tillin and Folland 2014; Roig et al. 2009), the earlier increase in MVT_ECC_ compared to MVT_ISO_ and MVT_CON_ might have been due to the larger negative muscle work and/or the percentage of time in negative muscle work (Buczek and Cavanagh 1990). However, no significant difference between contraction modalities was found regarding percentage MVT increases at 4 weeks, indicating that DR training improved MVT to a similar extent in all three contraction modalities tested.

The mechanisms underpinning strength improvements with eccentric training are thought to involve a combination of factors involving neural and muscle morphological and architectural adaptations (Douglas et al. 2017a). For the first time, we reported significant increases in muscle size (i.e. VL PCSA, volume and ACSA_mean_) following 4 weeks’ DR training, indicating that the chronic use of DR can stimulate muscle hypertrophy of the main locomotor muscles, i.e. quadriceps femoris. To ensure that the observed muscle adaptations were a consequence of muscle hypertrophy and not repeated measurement bias, intra-participant muscle adaptations were further observed using minimum detectable change measurements (Table 1). In fact, all participants’ adaptations exceeded the respective threshold for VL ACSA_mean_ and volume at 4 weeks, and 9 out of 12 exceeded the respective threshold for PCSA. Thus, the observed muscle adaptations could be interpreted as likely true muscle hypertrophy. This result is consistent with previous studies using high-intensity eccentric (Seynnes et al. 2007; Franchi et al. 2014) or plyometric (Monti et al. 2020) exercises, reporting muscle hypertrophy after just a few weeks of training. Moreover, we observed muscle hypertrophy after just 2 weeks of DR training regarding VL muscle volume and ACSA_mean_. Although results could be influenced by exercise-induced oedema (Damas et al. 2016), we suggest that its influence on the observed results is minimal, since a longer rest period (3–4 days) was respected between the last training session completed and the morphological muscle measurements. It should be emphasised that the present study is not the first to detect significant muscle hypertrophy after 10–14 days of eccentric training in previously untrained, healthy subjects (e.g. Seynnes et al. 2007; Stock et al. 2017), indicating that muscle morphological adaptations occur rapidly in response to chronic eccentric exercise. However, in contrast to the high forces and relatively few repetitions involved in the resistance studies by Stock et al. (2017) and Seynnes et al. (2007), the eccentric forces generated during the DR running were likely lower but the number of repetitions (i.e. strides) was much greater. Thus, it is possible that the overall volume of eccentric exercise was similar between studies. One could argue, therefore, that early muscle morphological and architectural adaptations could be related to the volume of eccentric exercise, rather than the intensity/load per se. Indeed, some of our pilot data suggest that participants were performing 160–170 strides per min at DR_15_, at a speed corresponding to 60–65% $\dot{V}$O_2max_. This means that participants were performing thousands of low-intensity eccentric contractions per training session. This may also partly explain the discrepancies observed with the study by Theofilidis et al. (2021), who reported decreases in VL muscle thickness and *L*_f_, and no change in PA after 8 weeks’ (two sessions a week) high-speed (90% of the speed associated with $\dot{V}$O_2max_) short-term interval (10 × 30 s; r’ = 60 s) DR training.

In the present study, muscle hypertrophy was associated with significant changes in muscle architecture. Increases in VL PA (+5.8%) and *L*_f_ (+2.7%) were detected after 4 weeks’ DR training. These findings are in line with previous studies reporting increases in VL PA and *L*_f_ after eccentric (Seynnes et al. 2007) and plyometric (Monti et al. 2020) training. While an increase in *L*_f_ has been suggested to reflect the addition of sarcomeres in series (Degens et al. 2009; Reeves et al. 2004b), a concomitant increase of PA may refer to the addition of sarcomeres in parallel (Degens et al. 2009; Aagaard et al. 2001). The addition of sarcomeres in series has been suggested to be an adaptive muscle mechanism in response to exercise-induced muscle damage (Proske and Morgan 2001). However, recent evidence suggests that increases in sarcomere length, rather than sarcomere number, may explain the increase in *L*_f_ observed following 3 weeks’ eccentric resistance training (Pincheira et al. 2021). Thus, as sarcomeres were not measured in the present study, further work is required to investigate the mechanisms underpinning the increases in VL *L*_f_ elicited by short-term DR training.

Strength improvements observed after a period of resistance training are also partly underpinned by adaptations of the central nervous system (for recent reviews Hortobágyi et al. 2021; Aagaard et al. 2020; Siddique et al. 2020; Pearcey et al. 2021; Skarabot et al. 2021). In the present study, VL EMG RMS but not VA or VL EMG RMS/M_max_ during MVT_ISO_ increased significantly after 4 weeks’ DR training. These results are partially consistent with previous reports, suggesting an increased agonist VA (Aagaard et al. 2000) and maximum VL EMG activity measured during MVT_ECC_ (Higbie et al. 1996; Hortobágyi et al. 1996) following 6 to 12 weeks *high-intensity*, *low- to moderate-volume* eccentric training. Moreover, whether neuromuscular adaptations were defined as changes in VL EMG RMS, VL EMG RMS/*M*_max_ or VA, neural changes appeared to contribute the most to changes in MVT_ISO_, at both 2 and 4 weeks in the current study. Although the ITT was only measured during MVT_ISO_, we correlated the percentage changes in VA with percentage changes in MVT during all three contraction modes. We found a very strong correlation with the change in MVT_ISO_ after 4 weeks and only a moderate correlation with the change in MVT_CON_ after 2 weeks. We propose that the lack of strong correlations between change in VA and change in MVT_ECC_ or MVT_CON_ is probably explained by the fact that VA was assessed only during MVT_ISO_ and/or that MVT_ISO_ is a more sensitive contraction mode to detect relationships between change in MVT and neural adaptations. Interestingly, VL muscle hypertrophy (in terms of both PCSA and volume) in the present study appeared to moderately contribute to MVT_CON_ changes but only during the latter half of the DR training period, while there were no correlations with changes in MVT_ISO_ or MVT_ECC_, at any time point. Since the calculation of muscle volume and PCSA involves *L*_f_ (representing the total number of sarcomeres in series), and *L*_f_ is the main determinant of muscle fibre maximal shortening velocity (Degens et al. 2009), this may partly explain why hypertrophy was related to changes in MVT during shortening, rather than isometric or lengthening contractions. It may also be possible that the unfamiliarity of maximal eccentric contractions performed on an isokinetic dynamometer may have precluded a relationship with neural and morphological adaptations. Indeed, while DR is characterised by repeated eccentric KE muscle contractions, they are performed at submaximal intensities and a relatively short muscle length. Finally, the relatively few correlations between morphological muscle changes and MVT changes could be partly explained by the short-term duration of the DR training programme. Indeed, morphological adaptations may have contributed to a greater proportion of MVT changes, had the DR training period been longer in duration. Nevertheless, the fact that we observed considerable changes in muscle size after 2 and 4 weeks, and that these changes correlated with changes in MVT_CON_, suggests that the eccentric nature of chronic DR is a potent stimulator of muscle hypertrophy and that these early increases in muscle size explain some of the MVT gains during the latter half of the short-term DR training period.

Interestingly, the present DR training did not significantly enhance maximal oxygen uptake in previously untrained, healthy adults. This result is consistent with previous studies reporting no change in $\dot{V}$O_2max_ in untrained (Toyomura et al. 2018) or endurance-trained (Shaw et al. 2018) individuals. It is likely that the relatively low metabolic intensity (i.e. 60–65% $\dot{V}$O_2max_) and/or training duration in the present study was not sufficient to promote change in maximal oxygen uptake. As training at or near to the velocity at $\dot{V}$O_2max_ appears to be the most effective way to enhance the maximal oxygen uptake (Midgley et al. 2006), it is not surprising that the present DR training did not induce such adaptations. Nevertheless, it is our opinion that DR training represents an effective method of stimulating muscle hypertrophy and strength gains, while simultaneously maintaining and/or challenging the cardiorespiratory system, a crucial advantage compared to traditional gym-based resistance training.

## Limitations

We acknowledge some limitations with the present study. First, the study did not include a control group, however, participants were instructed not to change their habitual physical activity and nutritional behaviour for the duration of the study. Moreover, participants were familiarised with the experimental procedures before data collection and the test–retest reproducibility of the measurements used to investigate neuromuscular adaptations was very high (i.e. all CVs were low except for MVT_ECC_, and all ICCs > 0.80 with narrow CIs except for VA, likely due to the data of just two participants). Thus, it is unlikely that the observed strength changes were due to measurement error or a learning effect. Second, participants included in the present study were not endurance-trained individuals and had no DR and/or eccentric-based training experiences. However, studying the effects of DR training on untrained individuals allows the detection of relatively larger changes in strength over a relatively short training period, which may not be possible in trained individuals. Moreover, while we acknowledge that the sample size could have been larger (three participants dropped out due to personal reasons), it still allowed us to draw firm conclusions about the benefit of DR training on young, healthy individuals, in terms of outlining the time course for neuromuscular adaptations in response to short-term DR training, and their specific relationships with strength gains. Furthermore, the total number of participants who completed the 4 weeks’ DR training and experimental tests exceeded the estimated required sample size, and was higher than the only other study that has reported significant strength gains following short-term DR training (*n* = 10; Toyomura et al. (2018).

## Conclusion

Rapid neuromuscular adaptations of the knee extensors were observed following 4 weeks’ DR training in previously untrained, healthy adults. Notably, gains in eccentric MVT occurred after just 2 weeks (+8.6%), while similar gains in eccentric, concentric and isometric strength occurred after 4 weeks (+9.7–15.2%). These functional changes were accompanied by changes in VL EMG activity after 2 and 4 weeks, while VL muscle hypertrophy could be seen as early as 2 weeks. These were followed by significant morphological (i.e. increased VL muscle volume and PCSA) and architectural (i.e. increased PA and *L*_f_) adaptations of the VL after 4 weeks’ DR. Changes in MVT (both at 2 and 4 weeks) were related predominantly to neural adaptations, while muscle hypertrophy was moderately related to changes in concentric MVT during the latter 2 weeks only. These novel data demonstrate how just 4 weeks of an ecologically valid mode of moderate-intensity chronic exercise can elicit neuromuscular adaptations in healthy young adults of the magnitude typically seen after a period of very high-intensity eccentric resistance training. However, the short training period and/or running intensity used was not sufficient to enhance aerobic capacity. Future studies should investigate if short-term, moderate-intensity DR can cause similar neuromuscular adaptations in both clinical and athletic populations.

## Abbreviations

ACSA: Anatomical cross-sectional area
ANOVA: Analysis of variance
CV: Coefficient of variation
DR: Downhill running
DR_5_: Downhill run at − 5% slope
DR_10_: Downhill run at − 10% slope
DR_15_: Downhill run at − 15% slope
EMG: Electromyography
ICC: Intraclass coefficient
ITT: Interpolation twitch technique
KE: Knee extensors
*L*_f_: Fascicle length
M_max_: Maximal M-wave amplitude
MT: Muscle thickness
MVT: Maximal voluntary torque
MVT_ISO_: Maximal voluntary isometric torque
MVT_ECC_: Maximal voluntary eccentric torque
MVT_CON_: Maximal voluntary concentric torque
PA: Pennation angle
VA: Voluntary activation
*r*: Pearson's correlation coefficient
VL: *Vastus lateralis*
$\dot{V}$O_2_ max: Maximal oxygen uptake
